# Distinct Metabolic Profiles of Ocular Hypertensives in Response to Hypoxia

**DOI:** 10.3390/ijms25010195

**Published:** 2023-12-22

**Authors:** Mia Langbøl, Jens Rovelt, Arevak Saruhanian, Sarkis Saruhanian, Daniel Tiedemann, Thisayini Baskaran, Cinzia Bocca, Rupali Vohra, Barbara Cvenkel, Guy Lenaers, Miriam Kolko

**Affiliations:** 1 Department of Drug Design and Pharmacology, University of Copenhagen, 2100 Copenhagen, Denmark; mia.langboel@sund.ku.dk (M.L.); jr.andreasen@sund.ku.dk (J.R.); arevak@sund.ku.dk (A.S.); sarkis.saruhanian@sund.ku.dk (S.S.); daniel@tiedemanns.dk (D.T.); baskaran.thisayini@gmail.com (T.B.); rvohra@sund.ku.dk (R.V.); 2 Department of Ophthalmology, Copenhagen University Hospital, Rigshospitalet, 2600 Glostrup, Denmark; 3 Department of Veterinary & Animal Sciences, University of Copenhagen, 2000 Frederiksberg, Denmark; 4 Faculté de Santé, Institut MITOVASC, UMR CNRS 6015, INSERM U1083, Université d’Angers, 49933 Angers, France; cinzia.bocca@univ-angers.fr (C.B.); guy.lenaers@inserm.fr (G.L.); 5 Département de Biochimie et Biologie Moléculaire, Centre Hospitalier Universitaire (CHU), 49933 Angers, France; 6 Department of Ophthalmology, University Medical Centre Ljubljana, 1000 Ljubljana, Slovenia; barbara.cvenkel@gmail.com; 7 Faculty of Medicine, University of Ljubljana, 1000 Ljubljana, Slovenia

**Keywords:** glaucoma, metabolomics, energy metabolism, oxygen stress, normal-tension glaucoma, ocular hypertension, hypoxia

## Abstract

Glaucoma is a neurodegenerative disease that affects the retinal ganglion cells (RGCs). The main risk factor is elevated intraocular pressure (IOP), but the actual cause of the disease remains unknown. Emerging evidence indicates that metabolic dysfunction plays a central role. The aim of the current study was to determine and compare the effect of universal hypoxia on the metabolomic signature in plasma samples from healthy controls (*n* = 10), patients with normal-tension glaucoma (NTG, *n* = 10), and ocular hypertension (OHT, *n* = 10). By subjecting humans to universal hypoxia, we aim to mimic a state in which the mitochondria in the body are universally stressed. Participants were exposed to normobaric hypoxia for two hours, followed by a 30 min recovery period in normobaric normoxia. Blood samples were collected at baseline, during hypoxia, and in recovery. Plasma samples were analyzed using a non-targeted metabolomics approach based on liquid chromatography coupled with high-resolution mass spectrometry (LC-HRMS). Multivariate analyses were conducted using principal component analysis (PCA) and orthogonal partial least squares-discriminant analysis (OPLS-DA), and univariate analysis using the Wilcoxon signed-rank test and false discovery rate (FDR) correction. Unique metabolites involved in fatty acid biosynthesis and ketone body metabolism were upregulated, while metabolites of the kynurenine pathway were downregulated in OHT patients exposed to universal hypoxia. Differential affection of metabolic pathways may explain why patients with OHT initially do not suffer or are more resilient from optic nerve degeneration. The metabolomes of NTG and OHT patients are regulated differently from control subjects and show dysregulation of metabolites important for energy production. These dysregulated processes may potentially contribute to the elevation of IOP and, ultimately, cell death of the RGCs.

## 1. Introduction

Glaucoma is an age-related, progressive, and debilitating disease that manifests in the eye. It is the leading cause of irreversible blindness worldwide [1]. With an increasingly aging population, the number of people affected by glaucoma is expected to rise from 64.3 million in 2013 to 111.8 million in 2040 [2]. The disease is defined by optic nerve degeneration accompanied by visual field defects. The condition affects the innermost nerve cells of the retina, known as the retinal ganglion cells (RGCs). The most common form of glaucoma is primary open-angle glaucoma (POAG), which presents in two subtypes defined by increased intraocular pressure (IOP), high-tension glaucoma, or IOP within the normal range, normal-tension glaucoma (NTG). Another group of patients presents with elevated IOP but no glaucoma. This subtype is called ocular hypertension (OHT). Risk factors for glaucoma include age, genetics, and elevated IOP [3]. Comorbidities such as hypertension, diabetes, and cardiovascular disease are common in glaucoma patients [4,5,6,7,8,9,10,11,12,13], suggesting that glaucoma may be associated with several systemic diseases. In this context, mitochondrial dysfunction, oxidative stress, and inflammation have been found to be involved in glaucoma pathogenesis [14,15,16,17]. Despite recognizing that there are many risk factors, current evidence-based treatments exclusively target IOP instead of directly protecting RGCs [18,19].

To better understand the pathophysiology of glaucoma, multiple mechanisms have been implicated in the loss of RGCs, including glutamate excitotoxicity, dysimmunity, altered inflammatory cytokines, and oxidative stress [16,20,21,22,23]. One potential and extensively investigated contributor is mitochondrial dysfunction [24,25,26]. Imbalances in the metabolic pathway can lead to impaired production and utilization of adenosine triphosphate (ATP) and other metabolites, as well as decreased clearance of reactive oxygen species (ROS), possibly exacerbating dysfunction and loss of RGCs [27]. Analyzing the metabolome in glaucoma patients is one way to investigate this possible imbalance and its associations with glaucoma.

Metabolomics is a powerful technique that combines small-molecule analysis with mathematical modeling to uncover the metabolic fingerprints of diseases and interventions. The technique offers distinct profiles closely linked to the phenotype of the disease by considering indirect factors such as age and lifestyle [28,29]. Despite its potential to uncover biomarkers, novel risk factors, and treatment options, metabolomics has not been widely applied to the study of glaucoma. To our knowledge, only a few studies have utilized metabolomics to analyze the blood of patients with glaucoma. One study used high-resolution mass spectrometry (HRMS) to compare the plasma metabolome of patients with POAG and healthy controls. This study revealed 41 discriminant metabolites that indicated alterations in blood fatty acid metabolism, steroid biosynthesis, and sphingolipid metabolism [30,31]. Another study identified N-lactoyl-phenylalanine as a potential and novel biomarker in plasma for POAG [31]. The results emphasize the importance of using metabolomics to gain a deeper understanding of glaucoma and identify potential biomarkers and therapeutic targets [32,33].

In this study, we aimed to examine the impact of systemic stress, and more specifically, universal mitochondrial stress, by exposing participants to hypoxia. The objective was to investigate the relationship between fluctuations in oxygen supply and oxidative stress, as well as energy metabolism. We compared the metabolic signatures of NTG and OHT patients with those of healthy controls and found that patients with OHT have uniquely regulated metabolites, mainly related to fatty acid metabolism, which may protect these patients from developing glaucoma despite high IOP.

## 2. Results

The characteristics of the study groups are listed in Table 1. Statistical analysis of pairwise comparisons between group characteristics confirmed that IOP was significantly higher in OHT patients than in controls and NTG patients in both eyes. The mean defect (MD) was found to be significantly higher in patients with NTG than in controls and patients with OHT in both eyes. There were no significant differences between the groups regarding age, body mass index (BMI), or sex.

Statistical comparisons:

- Age, BMI, and Sex: Ns
- IOP OD: Control vs. NTG (ns); Control vs. OHT (*p* < 0.0001); NTG vs. OHT (*p* < 0.0001)
- IOP OS: Control vs. NTG (ns); Control vs. OHT (*p* < 0.0001); NTG vs. OHT (*p* < 0.0001)
- MD OD: Control vs. NTG (*p* < 0.01); Control vs. OHT (ns); NTG vs. OHT (*p* < 0.05)
- MD OS: Control vs. NTG (*p* < 0.0001); Control vs. OHT (ns); NTG vs. OHT (*p* < 0.0001)

Data are presented as mean values (± SD) for continuous variables. The number of participants in each group is given as “N”. BMI: body mass index, IOP: intraocular pressure, MD: mean defect, NTG: normal-tension glaucoma, OD: oculus dexter, OHT: ocular hypertension, OS: oculus sinister, SD: standard deviation, ns: not significant. ^BMI calculated as weight (kg)/height^2^ (meter).

### 2.1. Metabolite Detection

A total of 136 metabolites were accurately detected using positive and negative ionization modes in addition to the seven internal standards. MS/MS fragmentation matching was possible for more than 85% of the detected metabolites. For the remaining metabolites, identification was based on isotopic pattern and retention time (RT). The most abundant class of molecules detected were amino acids, accounting for over 30%, followed by fatty acids, carbohydrates, and purines, each accounting for over 5% of the total. The remaining metabolites belonged to the families of keto acids, steroids, purine nucleosides, benzoic acids, and dicarboxylic acids.

### 2.2. Signatures of Hypoxia

**Figure 1:**
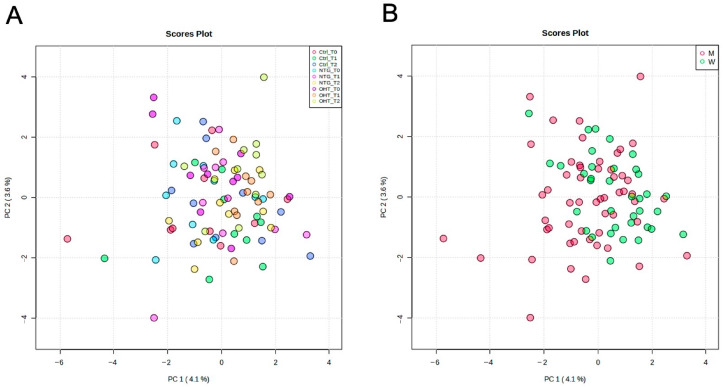
Principal component analysis (PCA) score plots of the full dataset and sex. (**A**): PCA score plot of controls, NTG, and OHT at baseline (T0), hypoxia (T1), and recovery (T2). (**B**): PCA score plot of all samples colored according to sex. Ctrl: control, M: men, W: women.

To identify underlying patterns in the metabolic data, we conducted a principal component analysis (PCA) to investigate the population structure. No spontaneous clustering was observed, and no confounding effect of sex distribution was highlighted (Figure 1A,B). PC1 and PC2 explain less than 8% of the variation, which is low. This observation can be attributed to several factors. First, the complexity of the dataset may be such that the variance is distributed across many components, reflecting a multifactorial structure with no dominant factors. Secondly, the small sample size may also contribute to this result. With fewer observations, the power to detect a strong pattern of variance along the first few components is reduced. It is also possible that the inherent noise in the data is more pronounced due to the small sample size, thereby diluting the variance captured by PC1 and PC2. Due to the small size and the heterogeneity of our population, orthogonal partial least squares-discriminant analysis (OPLS-DA) models failed to predict class membership.

**Figure 2:**
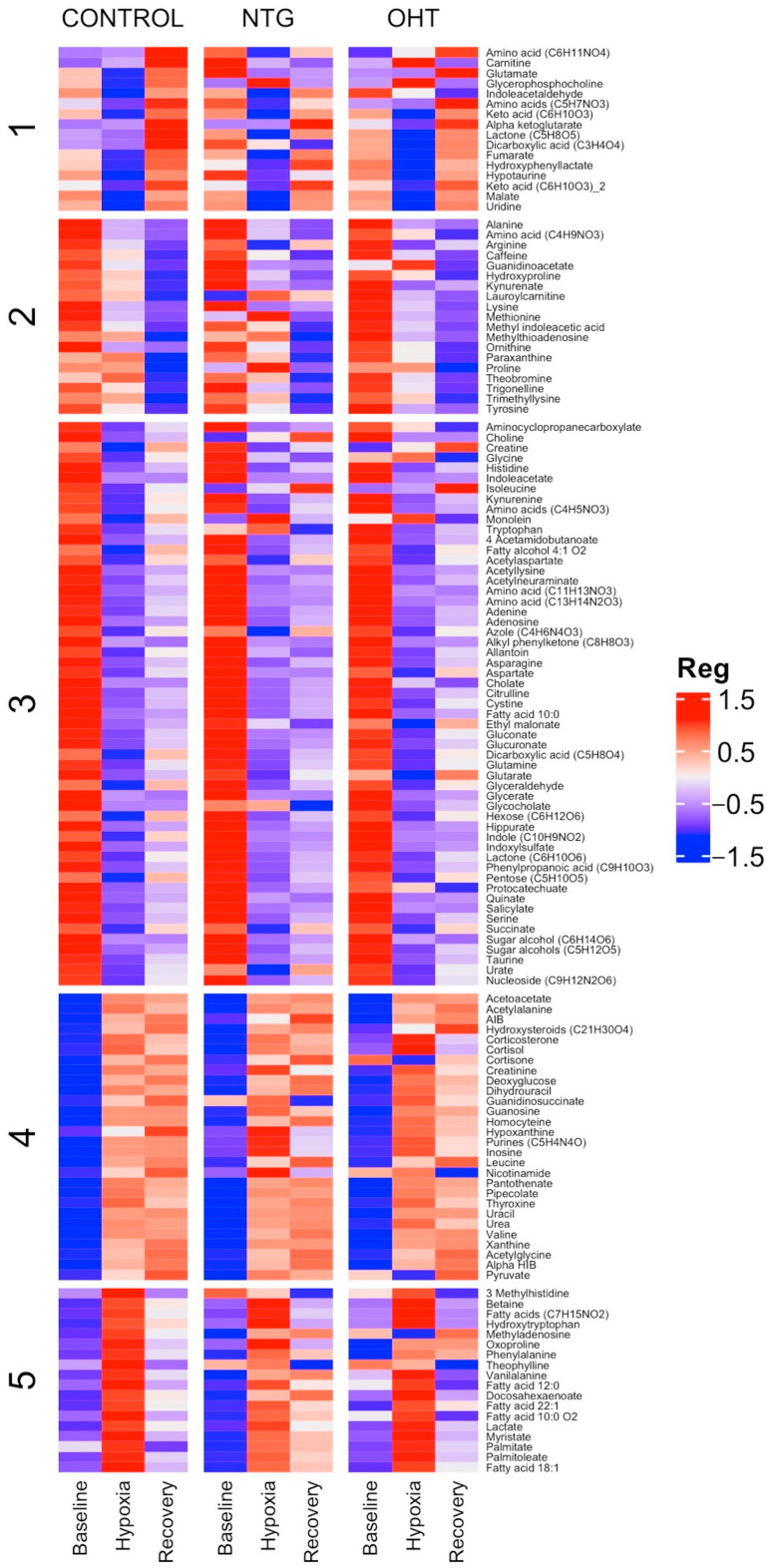
Heatmap showing the metabolome for controls, NTG, and OHT at baseline, hypoxia, and recovery. Clustering is based on the control group, and metabolite names are displayed as row labels. Reg: regulation.

To gain a better understanding of the data patterns, a heatmap was created to visualize the relationships among the three groups for all the identified metabolites (Figure 2). The heatmap is clustered according to the controls and indicates that the overall regulatory patterns are mostly consistent, with only a limited number of metabolites in the NTG and OHT groups displaying variations.

### 2.3. Baseline vs. Hypoxia

Table 2 provides a detailed summary of the results from both multivariate and univariate analyses of metabolite levels for the comparisons of control, NTG, and OHT at baseline vs. hypoxia. When comparing the metabolic profiles of controls, NTGs, and OHTs, both similarities and differences were observed. Across all groups, acetylaspartate, adenine, citrulline, and glutamine were downregulated. Alpha-hydroxyisobutyric acid (alpha HIB) was the only metabolite that was upregulated. Fewer metabolites were regulated in the NTG and OHT groups compared to the control group. Allantoin, azole (C_4_H_6_N_4_O_3_), gluconate, and indoleacetate were downregulated in the control group but not in the NTG and OHT groups. The OHT group had a unique metabolic profile with increased levels of acetoacetate, fatty acid 18:1, and myristate and decreased levels of acetylneuraminate and tryptophan. Furthermore, the OHT group lacked a regulation of cystine, dicarboxylic acid (C_5_H_8_O_4_), fatty alcohol 4:1 O_2_, glyceraldehyde, hexose (C_6_H_12_O_6_), pentose (C_5_H_10_O_5_), and serine, which were downregulated in both the control and NTG group. In contrast, the NTG group did not display any distinct regulation but rather lacked a response for acetyllysine, adenosine, amino acid (C_13_H_14_N_2_O_3_), asparagine, nucleoside (C_9_H_12_N_2_O_6_), succinate, and taurine, which were downregulated in the control and OHT group.

**Figure 3:**
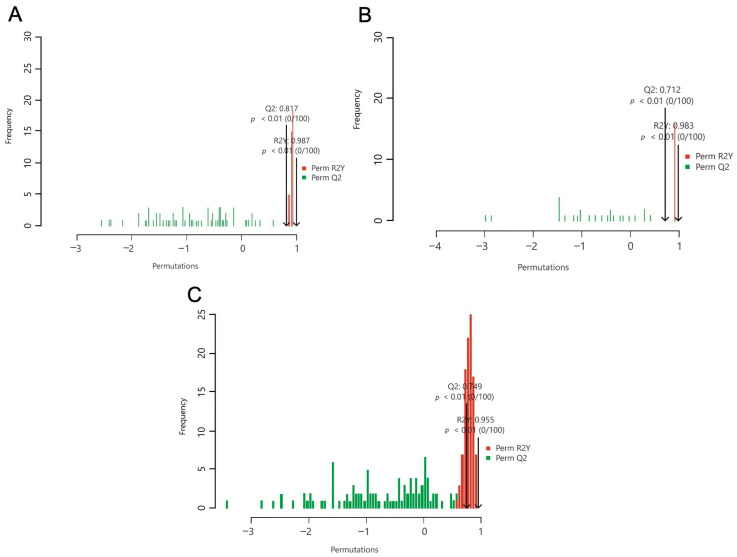
OPLS-DA permutation tests from baseline to hypoxia for metabolites in the control (**A**), NTG (**B**), and OHT (**C**) groups.

For the OPLS-DA models, the performance parameters were high, exhibiting both suitable model fit and prediction (control: Q2 = 0.749, *p* < 0.01, R2Y = 0.955, *p* < 0.01; NTG: Q2 = 0.817, *p* < 0.01, R2Y = 0.987, *p* < 0.01; OHT: Q2 = 0.712, *p* < 0.01, R2Y = 0.983, *p* = 0.02). Figure 3 illustrates the OPLS-DA permutation tests for each phenotype.

**Figure 4:**
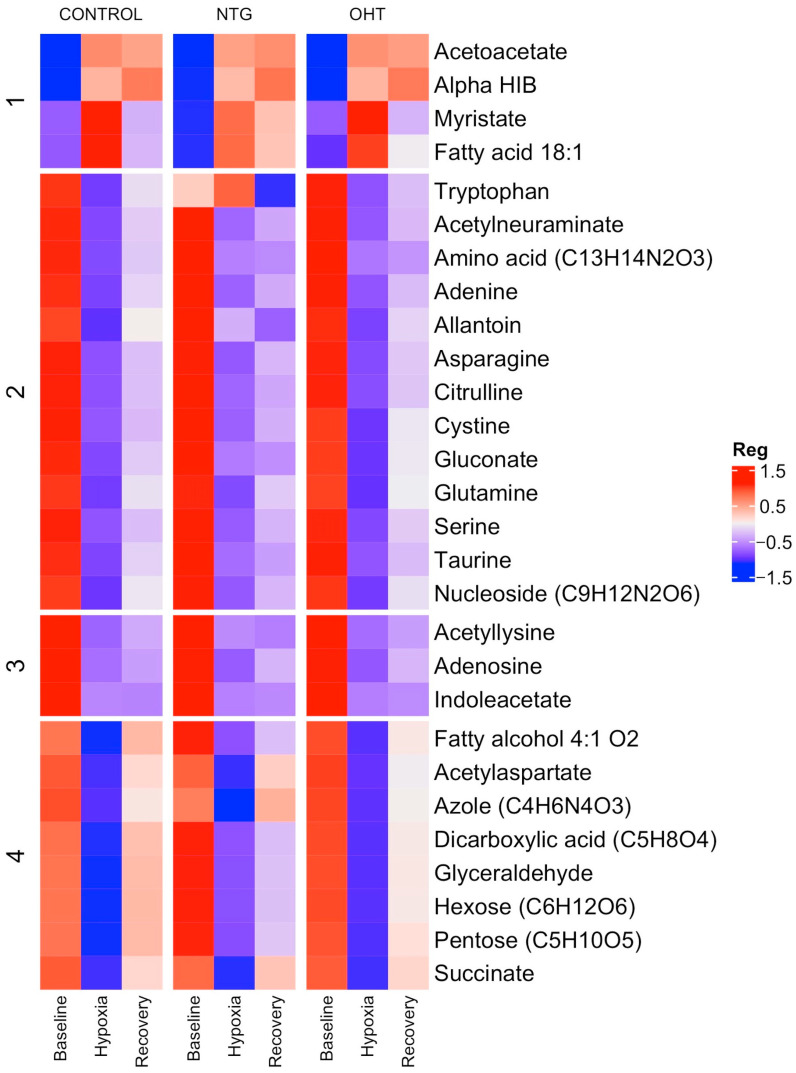
Heatmap showing significantly regulated metabolites in controls, NTG, and OHT at baseline, hypoxia, and recovery. The color scale, ranging from blue to red, indicates the average levels of the metabolites, with red representing high expression and blue representing low expression.

To visualize the differences among the regulated metabolites, a heatmap was made (Figure 4). Overall, the heatmap shows that the metabolites are similarly regulated across the groups, and any differences are likely to be specific to individual metabolites.

### 2.4. Hypoxia vs. Recovery

No metabolites were significantly regulated in the groups when comparing levels from hypoxia to recovery.

### 2.5. Time Point Comparisons

No significant differences were observed between the groups as a response to hypoxia. The OPLS-DA models did not produce noteworthy results, as they failed to accurately separate the different groups based on their metabolite profiles, and heatmaps displayed a consistent pattern of regulation across all groups and time points.

## 3. Discussion

The association between metabolic dysfunction and glaucoma is attracting increasing attention in the scientific community [23,34,35,36,37]. In this study, healthy control subjects and patients with NTG and OHT were subjected to oxygen stress by inducing hypoxia to evaluate their metabolic response. The findings indicate that NTG and OHT patients regulate certain metabolites differently in response to hypoxia than control subjects, which may be important for energy production and protection against stress. Patients with OHT appear to regulate metabolites that are not regulated by controls and NTG patients, which could offer protection. Metabolic dysfunction and reduced antioxidant capacity can lead to severe and potentially life-threatening effects on the body, such as cellular dysfunction, inflammation, and, ultimately, cell death. This has been observed in human and animal models of glaucoma, as well as in individuals experiencing prolonged hypoxia [17,24,38,39,40,41,42,43].

Studies have shown an enhanced systemic mitochondrial function and a strong antioxidant defense in patients with OHT [14,44]. It has, therefore, been hypothesized that OHT patients are protected from optic nerve degeneration. In the present study, unique metabolites were regulated in the OHT group compared to controls and NTG patients. Hence, we showed that acetoacetate, fatty acid 18.1, and myristate were upregulated, whereas acetylneuraminate and tryptophan were downregulated. Fatty acid 18:1 may refer to either petroselinic, petroselaidic, oleic, elaidic, or trans-vaccenic acid [45,46]. During limited oxygen availability, fatty acids are broken down into metabolites, including ketone bodies, that can be used as alternative sources of energy [47,48,49]. In addition, hypoxia may induce oxidative stress and inflammation, which can trigger elevation of fatty acids with antioxidant properties [46,50,51,52,53,54]. Elevated levels of ketone bodies and fatty acids could suggest enhanced energy production and a protective mechanism in OHT patients, supporting our previous findings of high antioxidant capacity and high levels of antioxidant lipid mediators [14].

The observed decrease in plasma tryptophan levels may be linked to changes in the kynurenine pathway. Tryptophan is mainly broken down through the kynurenine pathway, which donates electrons to the tricarboxylic acid (TCA) cycle and converts a significant portion of the tryptophan into various bioactive metabolites [55,56]. These metabolites are suspected to have antioxidant, anti-inflammatory, and/or neuroprotective properties [57,58,59,60]. Hypoxia could alter the activity of the kynurenine pathway, potentially affecting tryptophan metabolism and its downstream effects on improving immune function and neuronal health in OHT patients.

Hypoxia can cause damage to cells and tissues [61]. Acetylneuraminate is a sialic acid found in the cell membrane that plays a role in cell signaling, adhesion, and immunity [62]. It can protect cells and tissues from damage during hypoxia and repair damage that has already occurred. A possible explanation for the observed decrease in plasma levels of acetylneuraminate in OHT patients could be increased cellular uptake. Under hypoxic conditions, cells might upregulate the internalization and use of acetylneuraminate to modify glycoproteins and glycolipids on the cell surface [63]. This adaptive mechanism could serve to alter various cellular processes, including cell adhesion, signaling, and immune response, to offer protection and repair.

Although more research is needed, the results suggest that fatty acid metabolism may be upregulated in patients with OHT, as indicated by the upregulation of acetoacetate and fatty acids. Ketogenesis is a metabolic pathway that generates ketone bodies from fatty acids when carbohydrate or oxygen availability is low [64]. Research has suggested that ketone body and fatty acid metabolism may improve brain function, reduce inflammation, and protect against neuronal damage associated with certain neurodegenerative disorders, such as Alzheimer’s disease, Parkinson’s disease, Huntington’s disease, and glaucoma [65,66,67,68,69]. Additionally, studies have shown that alterations in the levels or composition of fatty acids in the brain may contribute to the development of these diseases by affecting cell function and structure [70,71,72]. Furthermore, our results suggest an increased activation of the kynurenine pathway, which could also imply that OHT patients have enhanced neuronal protection compared to NTG patients or healthy controls.

The NTG group displayed altered regulation of several metabolites, including acetyllysine, adenosine, asparagine, succinate, taurine, allantoin, gluconate, and indoleacetate. These metabolites were downregulated in controls but remained constant in NTG patients despite fluctuations in oxygen levels. Succinate, asparagine, and gluconate can be used for energy production in the TCA cycle [73,74]. Taurine is an amino acid that plays a role in supporting the TCA cycle. It acts as a buffer to stabilize the pH of the mitochondria and protects from oxidative stress damage [75,76]. Indoleacetate can come from the breakdown of tryptophan through the kynurenine pathway to support the TCA cycle and offer protection [77]. One could speculate whether NTGs are unable to use substrates for energy production to maintain energy levels during stress.

Adenosine can be released into the bloodstream to act as a neurotransmitter by binding to its receptors to slow down heart rate and dilate blood vessels during hypoxia. Adenosine is also released from cells when they are stressed or damaged and can offer protection by attracting immune cells to prevent further damage [78,79,80,81]. Allantoin is a biomarker for oxidative stress that can be triggered during hypoxia [82,83]. Acetylated lysine is a modified form of lysine that has been proposed to protect cells from stress damage, such as oxidative stress [84,85,86]. The role of acetylated lysine is not fully understood, however. Together, these results indicate that hypoxic intervention may trigger oxidative stress that is being actively counteracted in controls but not in NTG patients.

These results highlight a potential dysfunctional energy regulation, as compounds that would normally be taken up by the cells and used as alternative substrates for energy metabolism in the TCA cycle remain in the blood. Conditions of stress are also implicated that may cause the damage. These results are consistent with previous findings showing that amino acid levels in NTG patients remained unchanged despite changes in oxygen levels [87]. Impaired cellular uptake of substrates and mitochondrial dysfunction can cause a lack of energy in cells and organs, resulting in cellular dysfunction, oxidative stress, inflammation, and, ultimately, cell death [88,89]. Retinal neurons, in particular the RGCs, are more vulnerable when the body is exposed to stressors such as hypoxia since they are not myelinated and, therefore, require more energy to transmit a nerve signal and survive [34]. Although impaired transport of substrates into cells could be a plausible explanation for glaucoma, most research suggests that patients with NTG have impaired energy metabolism due to mitochondrial dysfunction in the RGCs [35,90,91]. We hypothesize that NTG patients have an increased universal mitochondrial vulnerability that manifests in the eye and the RGCs due to the high density of mitochondria. Dysregulation of oxidative stress and metabolites in conjunction with mitochondrial dysfunction has serious consequences for the body and leads to the development or exacerbation of neurodegeneration in glaucoma.

Patients with OHT also keep constant levels of acetyllysine, adenosine, asparagine, succinate, and taurine. Additionally, OHT patients maintain constant levels of cystine, glyceraldehyde, and serine, which were downregulated in controls. Serine can be converted into pyruvate, which is a key intermediate in the TCA cycle [92], and cystine can be used to produce taurine, which supports the TCA cycle [93]. Glyceraldehyde is an intermediate of glycolysis that can be used to synthesize triglycerides for energy production [94]. The lack of regulation in OHT may affect the production or drainage of aqueous humor elevation of IOP in these patients.

Acetylaspartate, adenine, alpha HIB, citrulline, and glutamine were regulated in all groups. All metabolites decreased except for alpha HIB, which increased during hypoxia. Acetylaspartate and glutamate may be used as substrates for energy production [95,96,97], and adenine may indicate an elevation of ATP production [41]. Citrulline is formed as a byproduct of nitric oxide (NO) production [98]. NO is a vasodilating compound that promotes oxygen delivery during hypoxia. Finally, alpha HIB is a compound known to increase during fasting [99,100]. The regulation of these metabolites indicates the general response to hypoxia exposure and suggests that our human model is effective.

In the identification of highly discriminating metabolites, we have reported the importance of orthogonal variables in the projection (O-VIP) values along with VIP scores. Although some O-VIP values exceeded the commonly used threshold of 1, these metabolites were kept for further analysis due to their significant VIP values, suggesting a substantial contribution to the predictive power of the model. Nevertheless, caution should be exercised when interpreting metabolites with O-VIP values marginally above 1. Elevated O-VIP scores may suggest that these metabolites capture variation that is orthogonal to the response variable, which may be due to structured noise or other systematic variations not directly related to the desired outcome. This observation does not necessarily undermine their importance but highlights the need for a balanced approach to data interpretation. Overall, the permutation tests for all three groups confirmed that the models were statistically robust and that the observed associations were not a result of random permutations of the data.

Analyses did not reveal any significant differences between controls, NTG, and OHT patients at either baseline, hypoxia, or recovery. This could be due to the limitations of the hypoxia model, the number of participants, and the methods used in the study. To uncover potential metabolic differences, the period of hypoxia might need to be extended, or the hypoxia would need to be more severe. However, this would be unethical and dangerous for the participants. Therefore, we suggest increasing the number of participants in the baseline studies to improve statistical power and to further investigate potential metabolic differences between groups in future studies. It could also be that metabolic regulation occurs mainly at the cellular level, and downstream products of intracellular energy metabolism are, therefore, not measurable in the blood. Further research using more sensitive and specific techniques may be necessary to explore in more detail the metabolic changes that occur in response to hypoxia.

## 4. Materials and Methods

### 4.1. Ethics

The current study was an interventional study that adheres to the guidelines outlined in the Declaration of Helsinki of 1975. It has received ethical approval from the National Committee on Health Research Ethics (project identification code: H-2-2014-060). Before enrollment, written informed consent for inclusion was obtained from each participant, and oral consent was obtained after a verbal explanation of the scope of the study.

### 4.2. Recruitment of Participants

A total of 30 individuals were recruited for the study between January 2017 and January 2020, divided into three groups of 10 individuals each. The groups consisted of patients diagnosed with NTG, OHT, and healthy controls. A total of 223 potential participants were allocated into 80 patients with OHT, 78 patients with NTG, and 65 control subjects were contacted for the study from 2017 to 2020. Eighteen patients with OHT, 15 patients with NTG, and 11 controls were excluded because they did not meet the inclusion and/or exclusion criteria. A total of 51 OHT patients, 45 NTG patients, and 36 controls declined to participate in the experimental setup after reading the participant information. The human experimental model was initiated with 12 patients with OHT, 18 patients with NTG, and 18 controls. In 1 OHT patient, 1 NTG patient, and 3 controls, the experimental setup was interrupted due to illness from altitude sickness. Hence, plasma samples were collected from a total of 11 OHT patients, 17 NTG patients, and 15 controls. After participating in the hypoxic setup, 1 OHT patient, 4 NTG patients, and 1 control were excluded because their visual field changed and thereby no longer met the inclusion and exclusion criteria. To have an equal number of participants in each group, 10 plasma samples from each group were analyzed. Patients with NTG and OHT were recruited from the Department of Ophthalmology at Rigshospitalet–Glostrup in Denmark, as well as from general ophthalmologists. Control subjects were recruited either from general ophthalmologists or as spouses of patients with NTG or OHT.

### 4.3. Inclusion Criteria

#### 4.3.1. NTG

Inclusion criteria for NTG were untreated IOP not exceeding 21 mmHg, measured at various times of the day from 8:00 AM to 5:00 PM, an open chamber angle on gonioscopy, glaucomatous optic nerve damage that violates the inferior ≥ superior ≥ nasal ≥ temporal (ISNT) rule [101], characteristic glaucomatous visual field loss as determined by Humphrey or Octopus perimetry, and significant nerve fiber loss observed by ocular coherence tomography (OCT).

#### 4.3.2. OHT

Inclusion criteria for OHT were untreated IOP greater than 24 mmHg, measured at various times of the day from 8:00 AM to 5:00 PM, an open chamber angle on gonioscopy, no optic nerve damage, a normal visual field by Humphrey or Octopus perimetry, and no significant nerve fiber loss observed by OCT.

#### 4.3.3. Control Group

Controls were participants with normal IOP and without any glaucomatous damage or visual field defects.

### 4.4. Exclusion Criteria

Participants with a history of ocular trauma or any other optic nerve or retinal nerve fiber disease other than glaucoma, significant systemic conditions such as severe and dysregulated hypertension, heart failure, hypercholesterolemia, diabetes mellitus, previous cerebrovascular events, or autoimmune diseases, and those who were unable to cope with the experimental setup were not eligible for enrollment. Mild cases of well-treated hypertension, hypercholesterolemia, and autoimmune diseases were acceptable. Moreover, participants below 50 years old and smokers were excluded.

### 4.5. Hypoxia Model and Plasma Collection

The method of inducing hypoxia used in this study has previously been described by Langbøl et al. and Vohra et al. [14,87]. Before the experiment, participants were instructed to fast for 10 h. Briefly, the participants were exposed to normobaric hypoxia for two hours using a tightly fitting facemask connected to a non-breathing valve to ensure that the participants were not breathing a hypercapnic gas mixture. The inhaled gas was a mixture of 10% oxygen and 90% nitrogen, which was supplied through the facemask connected by a Y-piece to a Douglas bag containing the humidified gas. After the two hours of hypoxia, the participants returned to normal breathing for 30 min in the supine position.

The experimental setup was designed to safely expose participants to hypoxia while minimizing the risk of complications or discomfort. The study was conducted in a laboratory setting with strict safety protocols, including continuous monitoring of vital signs such as heart rate, blood pressure, and oxygen saturation [14]. Participants were also followed with an electrocardiogram. If any discomfort was reported, the experiment was immediately stopped to ensure the safety of the participants.

To collect data from different phases of the experiment, blood samples were taken at three time points: before the onset of hypoxia (baseline), after two hours of hypoxia (hypoxia), and after 30 min of normoxia (recovery). A routine vein puncture was performed on the forearm. For each time point, 6 mL of whole blood was collected in a heparin tube, mixed thoroughly, and immediately placed on ice. Samples were processed within one hour of collection by centrifugation of the heparin tubes at 4000 RPM for 10 min at 4 °C. The plasma was aliquoted into Eppendorf tubes and stored at −80 °C for further analysis.

### 4.6. Chemicals and Reagents

Methanol (MeOH), water, and formic acid (Optima LC/MS grade) were acquired from Fisher Scientific (Illkirch, France). Isotope metabolite standards including 17α-Hydroxyprogesterone-d_8_ (2,2,4,6,6,21,21,21-d_8_), L-Thyroxine-^13^C_6_, Succinic acid-2,2,3,3-d_4_, Pyruvic acid-1-^13^C, and DL-Alanine-^15^N with >98% purity were acquired from Sigma Aldrich (St. Quentin Fallavier, France).

### 4.7. Metabolomic Analysis

We used a validated non-targeted reverse-phase (RP) method previously published [102,103], enabling the analysis of around 500 polar metabolites. Briefly, 30 µL of each plasma sample fortified with 10 µL of working standard solution (10 µg/mL in MeOH) was extracted with cold MeOH. After centrifugation, supernatants were evaporated to dryness and reconstituted in the mobile phase (aqueous solution with 2% MeOH). A total of 5 µL of each sample was injected randomly on Dionex UltiMate^®^ 3000 Ultra-High-Performance Liquid Chromatography (UHPLC, Dionex, Sunnyvale, CA, USA) equipped with an Acquity CSH C18 1.7 μm 150 mm × 2.1 column coupled with the precolumn CSH C18 1.7 μm VanGuard (Waters, Guyancourt, France), both kept at 40 °C. The detection was made in positive and negative mode on a Thermo Scientific Q Exactive HRMS (Thermo Fisher Scientific, Bremen, Germany) equipped with heated electrospray ionization (HESI II) after a multi-step gradient (mobile phase A: water with 0.1% formic acid; Mobile phase B: MeOH with 0.1% formic acid; flow rate: 0.3 mL/min; total runtime: 22.5 min).

Quality control (QC) samples, created by pooling the study population and processed in the same way as the other samples, were injected into each of the 10 samples. To ensure the reliability of the signals, QC diluted at 50% and 25% were also analyzed at the beginning and at the end of the sequence.

### 4.8. Sample and Metabolite Extraction

A TraceFinder 4.1 method (Thermo Fischer Scientific, Inc., Waltham, MA, USA) based on an in-house library was used for metabolite identification and peak integration. Data were filtered based on several criteria: a coefficient of variation (CV) in QC samples less than 30%, an *m*/*z* delta ppm less than 15, a coherent isotopic pattern, an RT drift fewer than 10 s, suitable linearity for dilutions with an R-squared value close to 1, and at least the match of 2 fragments in MS/MS spectra. If metabolites could not be identified using these criteria (level of metabolite identifications: 1, identified compounds) [104] but several similarities to data stored in our library were observed (i.e., fragmentation matching to another RT), molecules were named by their chemical formula and RT (level of metabolite identification: 2, putatively annotated compounds)^5^ with the belonging class where it was possible.

### 4.9. Statistical Power

Participants in the current study considerably overlap with participants in our previous studies using hypoxia as an intervention [14,87]. The power and sample size calculations for these studies were initially based on a previous study that measured arterial vessel diameters during hypoxia [105]. The calculations indicated that at least nine participants were required in each group, with a power of 80%, a *p*-value of 0.05, and an expected variation in diameters of 12%, using a mean arterial vessel diameter of 204.4 μm and a standard deviation of 18.6 from a parallel project. To confirm the power and sample size, we performed new calculations based on our previous study in which the metabolite lactate was measured in the plasma of controls during hypoxia exposure [87]. In this study, the mean lactate level was 1.1 ± 0.16 mmol/L and increased to 1.3 ± 0.09 mmol/L, an increase of 18%. With a power of 80% and a *p*-value of 0.05, these calculations indicated that at least ten participants were required in each group, validating the power of the current study.

### 4.10. Statistical Analyses

All analyses were performed using R (version 4.2.1, R Core Team, Vienna, Austria). Continuous variables were analyzed using one-way ANOVA (build-in function aov) and Tukey’s honest significant difference post hoc test (build-in function TukeyHSD), both of which are part of the base R statistical package. Categorical variables were analyzed using the chi-square test (build-in function chisq.test), also from the base R package [106]. With this methodology, we can determine whether there is a statistically significant difference in the data distribution among the compared groups. With the chi-square test, we examined the *p*-value to ascertain if there was a significant association between the categorical variables.

Raw metabolic data were initially normalized using the locally weighted scatterplot smoother (LOESS) regression function on galaxy.workflow4metabolomics.org to adjust for instrumental drift. To further minimize variations between individuals, normalization to the MS total useful signal (MSTUS) was applied using Microsoft Excel (Microsoft Corp., Redmond, WA, USA).

Data were further processed, using log_10_ transformation and Pareto scaling, and analyzed in MetaboAnalyst 4.0 (www.metaboanalyst.ca, accessed on 13 October 2022) [107]. Multivariate analysis, including PCA and OPLS-DA, was performed with 100 permutation tests. These analyses aimed to identify patterns and variations in the metabolite data and potential biomarkers. Univariate analysis was performed using the Wilcoxon signed-rank test with false discovery rate (FDR) correction to identify differentially expressed metabolites among different groups. To determine the magnitude of change, we calculated fold change (FC) values as the ratio of the mean levels of a given metabolite between different time points or groups, allowing us to identify metabolites with significant changes and quantify the extent of these changes.

Heatmaps were generated in R (version 4.2.1) [106] using the “ComplexHeatmap” library [108]. The clustering method used for the heatmaps was k-means. For the OPLS-DA models, the Q2 value evaluates the model’s predictive accuracy, with higher values indicating better performance. R2Y measures the model’s ability to separate samples into different groups and ranges from 0 to 1, with 1 being a perfect separation and values above 0.5 considered suitable. The cumulative Q2, Q2 (cum), evaluates the overall goodness-of-fit, with values above 50% indicating a strong explanation of data variation. The VIP value assigns importance to each variable in the model. The O-VIP value quantifies the contribution of each variable to the variation orthogonal to the predictive components, aiding in the identification of metabolites less likely to be related to the dependent variable when values exceed 1. Highly discriminant metabolites were selected based on the following criteria: OPLS-DA with Q2 *p* < 0.05 and R2Y *p* < 0.05, Q2 (cum) > 50%, *p* (corr) > 0.4, VIP value > 1, and fold change FDR < 0.05.

## 5. Conclusions

In summary, we observed the dysregulation of important metabolites involved in energy production in both NTG and OHT patients, as they regulated their metabolome differently than the control group. This dysregulation may be associated with the dysfunction and loss of RGCs observed in glaucoma. Interestingly, OHT patients exhibited regulation of unique metabolites involved in fatty acid biosynthesis, ketone body metabolism, and the kynurenine pathway, which are believed to play a role in neuroprotection. These results are in accordance with our previous study, in which we showed that patients with OHT have high antioxidant capacity and high levels of antioxidant lipid mediators compared to control subjects and patients with NTG. Overall, our findings suggest that metabolic dysregulation may be a pivotal factor in the development and progression of glaucoma. Further research is required to better understand the relationship between the presence and levels of specific metabolites, mitochondrial function, hypoxia, and glaucoma.

## Figures and Tables

**Table 1:** Demographic and clinical data for the three groups.

	Control	NTG	OHT
N	10	10	10
Age (years)	67 (8.1)	73 (6.8)	72 (4.3)
BMI	27.3 (7.1)	24.0 (2.6)	25.3 (2.4)
Sex (men/women)	7/3	6/4	5/5
IOP OD (mmHg)	13.6 (1.8)	12.1 (2.0)	29.3 (5.7)
IOP OS (mmHg)	13.6 (2.0)	12.5 (2.4)	31.2 (5.5)
MD OD (dB)	0.9 (1.6)	7.2 (7.0)	1.4 (1.7)
MD OS (dB)	1.0 (2.6)	11.1 (6.4)	0.9 (1.4)

**Table 2:** Metabolites that showed a statistically significant change between baseline and hypoxia. The table lists all metabolites that were differentially expressed in each group from baseline to hypoxia based on the results of orthogonal partial to least squares-discriminant analysis (OPLS-DA) and univariate analyses. For some metabolites, the molecular formula and identification information, in brackets, were determined based on the isotopic pattern and retention time (RT). Alpha HIB: alpha-hydroxyisobutyric acid, FC: fold change, FDR: false discovery rate, O-VIP: orthogonal variable importance in the projection, VIP: variable importance in the projection.

	Control				NTG				OHT			
Metabolite	FC	FDR	VIP	O-VIP	FC	FDR	VIP	O-VIP	FC	FDR	VIP	O-VIP
Acetylaspartate	0.7105	0.0278	1.7095	0.6008	0.7641	0.0258	0.8629	0.6428	0.7239	0.0119	1.7776	0.9227
Adenine	0.7149	0.0029	1.9385	0.6912	0.7845	0.0110	1.9661	0.7799	0.7948	0.0119	1.8624	0.9491
Alpha HIB	1.7137	0.0110	1.8708	0.6450	1.5854	0.0083	2.1538	0.4206	1.6238	0.0119	1.9119	0.6514
Citrulline	0.6038	0.0074	1.8991	0.8186	0.6123	0.0326	1.8805	0.8689	0.6678	0.0119	1.9123	0.8883
Glutamine	0.7188	0.0070	1.9642	0.9315	0.7405	0.0044	2.3512	0.7687	0.7627	0.0119	1.7434	0.9683
Allantoin	0.6548	0.0186	1.7486	0.6592								
Azole (C_4_H_6_N_4_O_3_)	0.8270	0.0261	1.6694	0.8197								
Gluconate	0.8071	0.0405	1.6574	0.7318								
Indoleacetate	0.5937	0.0278	1.5122	1.0439								
Acetoacetate									3.2305	0.0119	1.8952	0.4330
Acetylneuraminate									0.8243	0.0119	1.8315	0.9941
Fatty acid 18:1									1.3764	0.0189	1.7271	1.0603
Myristate									1.3488	0.0119	1.9050	0.5487
Tryptophan									0.8775	0.0103	2.0147	0.4868
Cystine	0.6916	0.0070	1.8868	0.0800	0.6626	0.0044	2.2478	0.6675				
Dicarboxylic acid (C_5_H_8_O_4_)	0.8970	0.0405	1.5093	1.0341	0.8358	0.0083	2.0548	0.6598				
Fatty alcohol 4.1 O_2_	0.8933	0.0278	1.5150	1.0399	0.8319	0.0083	2.0977	0.6549				
Glyceraldehyde	0.8881	0.0405	1.5000	1.0871	0.8189	0.0044	2.1532	0.6640				
Hexose (C_6_H_12_O_6_)	0.8961	0.0261	1.4972	0.9774	0.8320	0.0083	2.1303	0.6301				
Pentose (C_5_H_10_O_5_)	0.8840	0.0278	1.6628	1.0000	0.8280	0.0044	2.1664	0.6613				
Serine	0.7051	0.0219	1.6542	1.0954	0.7576	0.0205	1.8690	1.0349				
Acetyllysine	0.6862	0.0186	1.7629	1.1218					0.7376	0.0119	1.8707	0.8031
Adenosine	0.8066	0.0405	1.5172	1.0711					0.7683	0.0119	1.7795	0.8201
Amino acid (C_13_H_14_N_2_O_3_)	0.5119	0.0219	1.6143	0.5305					0.5210	0.0157	1.5710	0.9532
Asparagine	0.6709	0.0095	1.7561	1.1434					0.7338	0.0311	1.7116	0.9124
Nucleoside (C_9_H_12_N_2_O_6_)	0.8357	0.0110	1.8266	0.4729					0.8461	0.0189	1.7018	0.9529
Succinate	0.6415	0.0074	1.8306	0.6296					0.6895	0.0119	1.9347	0.9058
Taurine	0.7413	0.0029	2.0438	0.3861					0.7927	0.0245	1.5723	0.9030

## Data Availability

The data presented in this study are available on request from the corresponding author. The data are not publicly available because they contain information that could compromise the privacy of research participants.
